# Ovatodiolide targets chronic myeloid leukemia stem cells by epigenetically upregulating hsa-miR-155, suppressing the BCR-ABL fusion gene and dysregulating the PI3K/AKT/mTOR pathway

**DOI:** 10.18632/oncotarget.23231

**Published:** 2017-12-14

**Authors:** Yue-Xing Tu, Shi-Bing Wang, Luo-Qin Fu, Shuang-Shuang Li, Qian-Peng Guo, Yi Wu, Xiao-Zhou Mou, Xiang-Min Tong

**Affiliations:** ^1^ Department of Critical Care Medicine, Chun’an First People’s Hospital (Zhejiang Provincial People’s Hospital Chun’an Branch), Hangzhou 311700, Zhejiang Province, China; ^2^ Department of Critical Care Medicine, Zhejiang Provincial People’s Hospital, People’s Hospital of Hangzhou Medical College, Hangzhou 310014, Zhejiang Province, China; ^3^ Clinical Research Institute, Zhejiang Provincial People’s Hospital, People’s Hospital of Hangzhou Medical College, Hangzhou 310014, Zhejiang Province, China; ^4^ Key Laboratory of Tumor Molecular Diagnosis and Individualized Medicine of Zhejiang Province, Hangzhou 310014, Zhejiang Province, China; ^5^ Department of Hematology, Zhejiang Provincial People’s Hospital, People’s Hospital of Hangzhou Medical College, Hangzhou 310014, Zhejiang Province, China

**Keywords:** CML, hematopoietic stem cells, CD34^+^/CD38^−^, PI3K/AKT/mTOR, diterpenoid ovatodiolide

## Abstract

Chronic myeloid leukemia (CML) is a myeloproliferative pathology, originating from the hematopoietic cancer stem cells (hCSCs) due to the Bcl-Abl Philadelphia chromosome transformation. However, targeting these hCSCs as an effective anti-CML strategy is relatively less explored. Ovatodiolide (Ova) is a natural diterpenoid isolate of *Anisomeles indica* with broad anticancer activity. In this study, we investigated the anti-hCSCs potential of Ova against CD34^+^/CD38^−^, CD34^+^/CD38^+^, and unsorted K562 cell lines using flow cytometry, western blot, RT-PCR, genomic mapping, and tumorsphere formation assays. We demonstrated that compared to unsorted K562 and CD34^+^/CD38^+^, CD34^+^/CD38^−^ cells were significantly enriched with Oct4, Sox2, CD133, Bcr-Abl, p-CrkL and p-Stat5 protein and/or mRNA. Furthermore, we showed that Ova alone or by enhancing the therapeutic potential of Imatinib, reduced the viability of CML cell lines, dose-dependently, irrespective of the cancer stemness, as well as markedly inhibit the Bcr-Abl, p-CrkL, Stat5, and MDR protein expression levels in CD34^+^ cells. Mechanistic investigations revealed a significant up-regulation of hsa-miR-155, which resulted in the reduction of dysregulating the PIK3CA expression in Ova-treated K562 CD34^+^/CD38^−^ cells. Additionally, Ova alone or in combination with Imatinib suppressed the hCSC traits of the CD34^+^/CD38^−^ cells, resulting in loss of their ability to form tumorspheres, enhanced apoptosis, increase in the Bax/Bcl-2 ratio, and dysregulation of the PI3K/AKT/mTOR signaling pathway. Together, these results demonstrate the PI3K/AKT/mTOR signaling-mediated anti-hCSC effect of Ova in CML, as well as suggest a likely role for Ova as a small molecule PI3K/mTOR dual inhibitor, thus, extending its potential benefit to other mTOR-mediated pathologies.

## INTRODUCTION

Chronic myeloid leukemia (CML) accounts for about 15% of all leukemia in the developing world [1], and though it is not restricted to any age, CML is predominantly an adult disease, accounting for 20% of all leukemia in adults, with an estimated incidence of 1.6 per 100,000 in U.S alone, and 5430 diagnosed new cases in 2012 [2]. CML is a particularly aggressive and lethal myeloproliferative pathology, originating from the hematopoietic cancer stem cells (hCSCs) due to Bcl-Abl tyrosine kinase - mediated transformation of primitive hematopoietic cells and formation of the Philadelphia (Ph) chromosome. The role of hCSCs in tumor progression, metastasis, chemoresistance and recurrence in CML is well documented; however, targeting these hCSCs as an effective anti-CML strategy is relatively less explored [3].

Despite our current limited knowledge of the specific oncogenic events that initiate and/or sustain CML, CML remains a paradigm for hematopoietic stem cell (hSC)-derived malignancy, and the implication of hCSCs in the initiation, drug-resistance, distant metastases and recurrence of CML is increasingly becoming a focus of anticancer research [4, 5].

The small molecule inhibitor of Bcr-Abl kinase, Imatinib mesylate (IM), with demonstrated induction of complete cytogenetic and hematologic response in most chronic phase CML patients is the current standard of care for Ph^+^ CML [6], however, unfortunately, IM does not provide definitive cure to CML patients because of the development of resistance to IM by the Bcr-Abl fusion gene, as well as the primary insensitivity of hCSCs to IM [7]. This is also true of the other Bcr-Abl kinase inhibitors such as nilotinib and dasatinib, which have been shown to be ineffective in CML harboring BCR-ABL-T315I mutation, thus, rendering the therapeutic promise of IM and these other Bcr-Abl kinase inhibitors elusive in CML. In addition, STAT5 has been shown to contribute to two hallmarks for tumor initiation and malignant progression [8]. First, STAT5 can override the STAT3-mediated cell death program in the normal mammary epithelium. In addition, elevated STAT5 expression has been detected in subset of primary human breast cancers [9] and the constitutive STAT5 activation in luminal epithelial cells and multipotent progenitors is sufficient to initiate breast tumorigenesis [10]. More importantly, the activation of STAT5 through JAK2 has been demonstrated to be required for the initiation of mammary tumors *in vivo* [11]. It has been well established that downstream of Bcr-Abl, STAT5 functions to prevent apoptosis in CML cells. Together, targeting Brc-Abl/STAT5 axis may represent a more effective approach for CML interventions.

Hematopoietic CSCs, harbor stem cell-like properties, namely, high expression of stemness markers, self-renewal, initiation and maintenance of cancer, and resistance to anticancer treatment, thus constitute potential molecular targets for screening novel anti-CML drugs. Previously, Bamodu *et al.* demonstrated that Ovatodiolide (Ova) sensitizes triple negative breast cancer cells to doxorubicin and eliminates their CSC-like phenotype [12], and this was corroborated by Lu *et al.* who demonstrated that the inhibition of breast cancer stem/progenitor cells by Ova was through a SMURF2-mediated downregulation of Hsp27 [13]. Ova is a bioactive macrocyclic diterpenoid compound purified from the traditional Chinese herbal medicine Anisomeles indica (L.) Kuntze, with documented antiinflammatory, antiangiogenesis, antibacterial, antiviral, antioxidant, and anticancer activities [14, 15], however, nothing is known about the therapeutic effect of Ova in hematologic cancers, more so, regarding their ability to target and/or inhibit the CD34^+^/CD38^−^ hCSCs, therefore, in this study, we investigated the microRNA (miRNA)-mediated epigenetically therapeutic effect of Ova in the CML hCSCs and its underlying molecular mechanism.

miRNAs are small non-coding RNAs that are actively involved in the modulation of transcription factors and gene expression by binding to the 3′untranslated regions (3′UTRs) of the target messenger RNA (mRNA) to repress them [16]. miRNA expression has also been shown to change during the differentiation of certain hematopoietic stem cell (hSC) lineages [17]. In fact, animal models with silenced or ectopic expression of certain miRNAs have been used to demonstrate that miRNAs play very important role in the erythropoietic, granulopoietic, and monocytopoietic process, as well as in the development of T-lymphocytes [18, 19]. This is further corroborated by the implication of altered miRNA expression in hematologic cancers, howbeit more in mice models [20].

In this study, we report the hsa-miR-155-mediated Ova suppression of CD34+/CD38^−^ CML cell viability and hCSC-like phenotype, including their associated pluripotency and survival signaling, through the dysregulation the PI3K/AKT/mTOR signaling pathway.

## RESULTS

### CD34^+^/CD38^−^ CML cells exhibit enhanced cancer stem cell-like phenotype

**Figure 1:**
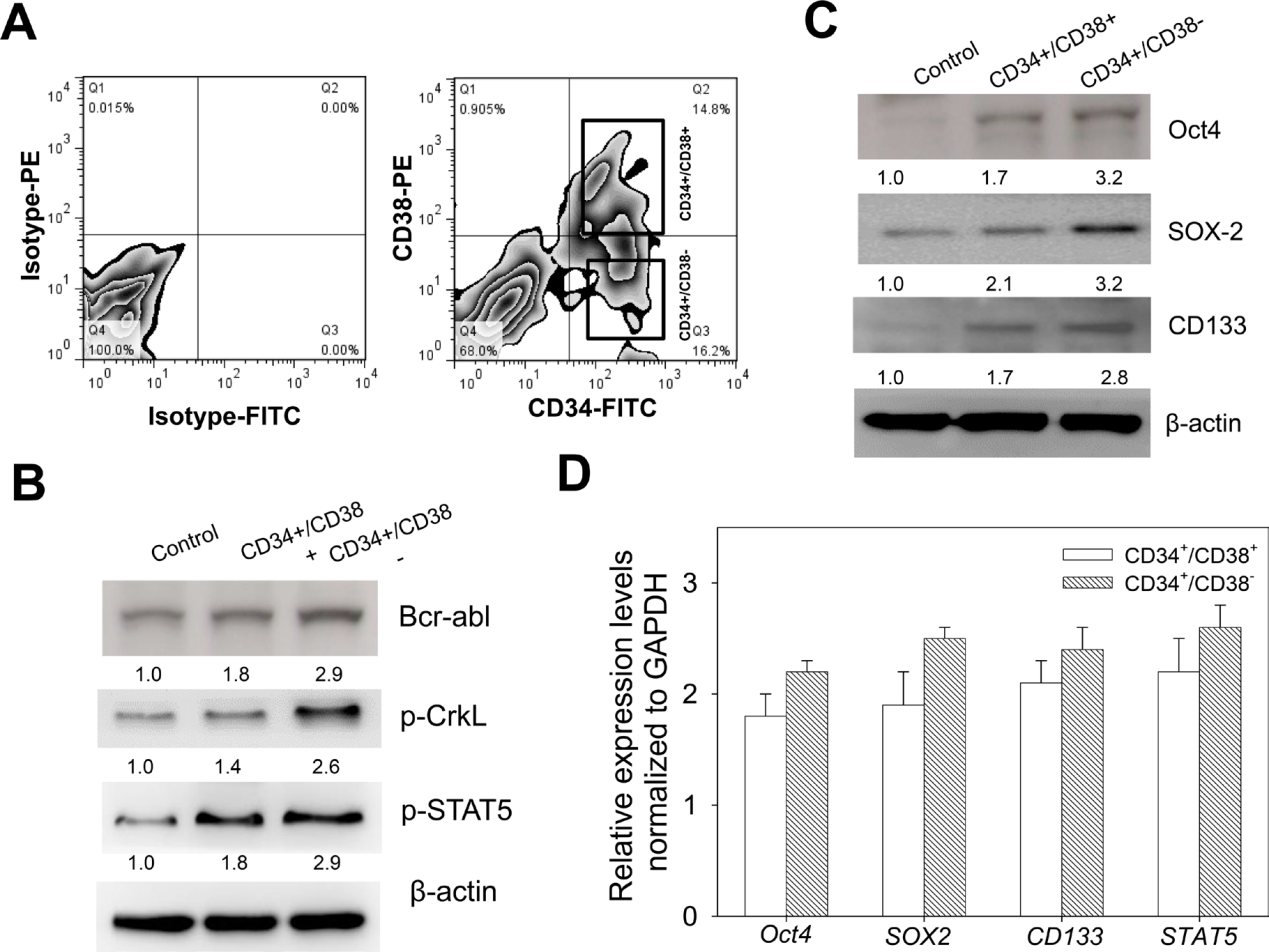
CD34^+^/CD38^−^ CML cells exhibit enhanced cancer stem cell-like phenotype (**A**) Flow cytometry-based cell sorting of K562 cells stained with Isotype-PE, Isotype-FITC, CD38-PE, and CD34-FITC, into CD34^+^38^–^ and CD34^+^38^+^ populations. (**B**) Analysis of the protein expression levels of Bcr-abl, p-CrkL and p-Stat5 in CD34^+^38^−^ or CD34^+^38^+^ K562 cells, compared to control unsorted K562 cells. (**C**) Western blot analysis of the expression profile of Oct4, Sox2 and CD133 proteins in CD34^+^38^−^ or CD34^+^38^+^ K562 cells, compared to control unsorted K562 cells. (**D**) Relative mRNA expression levels of Oct4, Sox2, CD133 and Stat5 normalized to GAPDH, using RT-PCR. β-actin was used as loading control.

To characterize the CML cell line K562, we sorted the cells into CD34^+^/CD38^−^ and CD34^+^/CD38^+^ populations using the FACS-based regions, after staining the cells with CD38-PE and CD34-FITC, with Isotype-PE and Isotype-FITC as controls. Based on our FACS data, we obtained 0.90% CD34^−^/CD38^+^, 14.8% CD34^+^/CD38^+^, 16.2% CD34^+^/CD38^−^, and 68.0% CD34^-^/CD38^−^, in Q1, Q2, Q3 and Q4, respectively (Figure 1A). Based on our understanding that CML patients harbor a consistently detectable population of quiescent Ph^+^ and Bcr-Abl^+^ CD34^+^ cells which are associated with constitutive activation of Stat5 and CrkL [21], we examined the expression profile of these CML-driving proteins in the K562 cells. Results of our western blot analyses indicate that Bcr-Abl, p-CrkL and p-Stat5 were all up-regulated in the CD34^+^/CD38^−^ cells, compared to the CD34^+^/CD38^+^ and unsorted K562 cells (Figure 1B). To understand this differential protein expression in the context of the quiescent self-renewing hematopoietic cell population, we further assessed the enrichment of the K562 cells with stemness factors, and demonstrated that the compared to the CD34^+^/CD38^+^ and unsorted K562 cells, the CD34^+^/CD38^−^ cells showed enhanced expression of the stemness markers Oct4, Sox2 and CD133 on the protein and mRNA levels (Figure 1C and 1D). These data indicate that CD34^+^/CD38^−^ cells exhibit enhanced hCSC-like phenotype.

### Ovatodiolide potentiates the anti-proliferative potential of Imatinib in CD34^+^ CML cells through disruption of the BCR^-^ABL signaling

**Figure 2:**
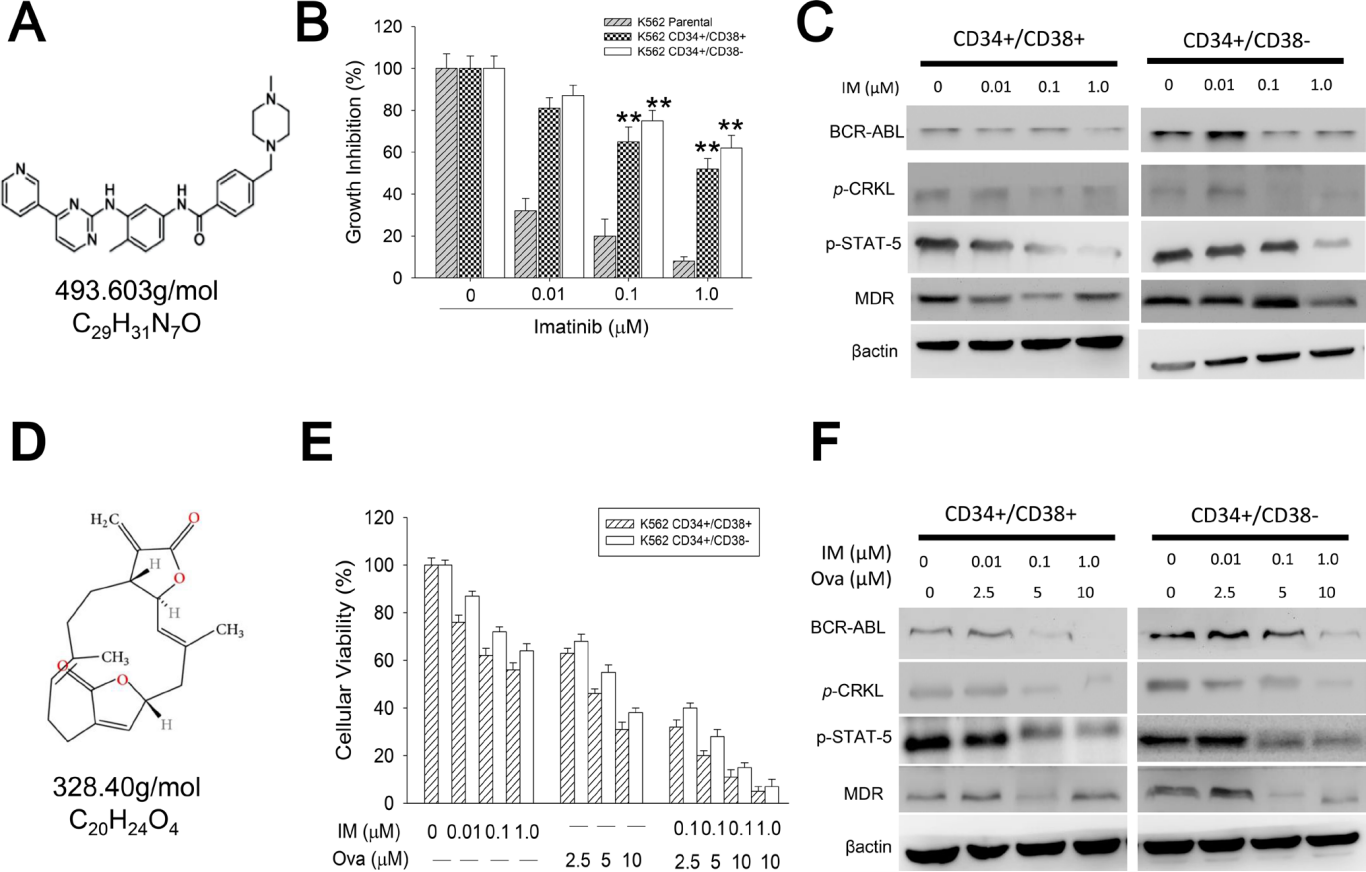
Diterpenoid ovatodiolide potentiates the anti-proliferative potential of Imatinib in CD34^+^ CML cells through disruption of the BCR-ABL signaling (**A**) The chemical structure of Imatinib, with molecular formula C29H31N7O and molar mass 493.603 g/mol. (**B**) The effect of 0.01–1.0 μM Imatinib on the cell viability of CD34^+^38^−^ or CD34^+^38^+^ K562 cells, compared to unsorted parental K562 cells. (**C**) Western blot analyses of the effect of 0.01–1.0 μM Imatinib on the protein expression levels of Bcr-abl, p-CrkL, Stat5, and MDR in CD34^+^38^−^ or CD34^+^38^+^ K562 cells. (**D**) The chemical structure of Ova, with molecular formula C20H24O4 and molar mass 328.408 g/mol. (**E**) The effect of 0.01–1.0 μM Imatinib and/or 2.5–10 μM Ova on the cell viability of CD34^+^38^−^ or CD34^+^38^+^ K562 cells. (**F**) Western blot analyses of the effect of 0.01–1.0 μM Imatinib and/or 2.5–10 μM Ova on the protein expression levels of Bcr-abl, p-CrkL, Stat5, and MDR in CD34^+^38^−^ or CD34^+^38^+^ K562 cells. β-actin was used as loading control. ^*^P < 0.05, ^**^P < 0.01, ^***^P < 0.001.

Since more than 90% of CML cases harbor the Bcr-Abl (t 9;22) chromosomal translocation and have been shown to respond to some degree to IM, a selective inhibitor of the Bcr-Abl tyrosine kinase, to understand how the cytogenetic classification of CML cells may affect their response to chemotherapy, we examined the effect of IM on the unsorted and sorted K562 cells. Using the cytotoxicity assay, we demonstrated that while IM (Figure 2A) significantly inhibited the cell viability of the unsorted K562 cells and moderately affected viability of the CD34^+^/CD38^+^ cells, it exhibited a 9%, 26% and 35% inhibitory effect on the viability of the CD34^+^/CD38^−^ cells, at a concentration of 0.01, 0.1, and 1.0 μM, respectively (Figure 2B). This was associated with dose-dependent down-regulation of the expression levels of Bcr-Abl, p-CrkL, p-Stat5 and MDR proteins in both CD34^+^/CD38^+^ and CD34^+^/CD38^−^ after 48 h IM treatment, however, this was more apparent in the CD34^+^/CD38^+^ cells (Figure 2C), invariably highlighting the inherent reduced sensitivity of the CD34^+^/CD38^−^ hCSC-like cells to IM treatment. Having demonstrated in other previous work that Ova is effective against CSCs in solid tumors [12, 13], we investigated its effect in the CML cells. Our results revealed that Ova (Figure 2D) significantly increased the sensitivity of the CD34^+^/CD38^−^ hCSC-like cells to IM treatment, as demonstrated by 60%, 69%, 84%, and 92% reduction in the cell viability of the of the CD34^+^/CD38^−^ hCSC-like cells after treatment with 0.1, 0.1, 0.1 or 1.0 μM IM in combination with 2.5, 5.0, 10.0, or 10.0 μM Ova, respectively (Figure 2E), and this was associated with the enhanced dose-dependent reduction of the expression levels of Bcr-Abl, p-CrkL, p-Stat5 and MDR proteins in both CD34^+^/CD38^+^ and CD34^+^/CD38^−^ cells (Figure 2F). These data do indicate that Ova potentiate the anti-proliferative potential of Imatinib in the CD34^+^ hCSC-like cells by disrupting the Bcr-Abl signaling.

### Ovatodiolide alone or in combination with Imatinib induce apoptotic cell death in CD34^+^CD38^−^ CML cells by dysregulating the PI3K/AKT/mTOR pathway

**Figure 3:**
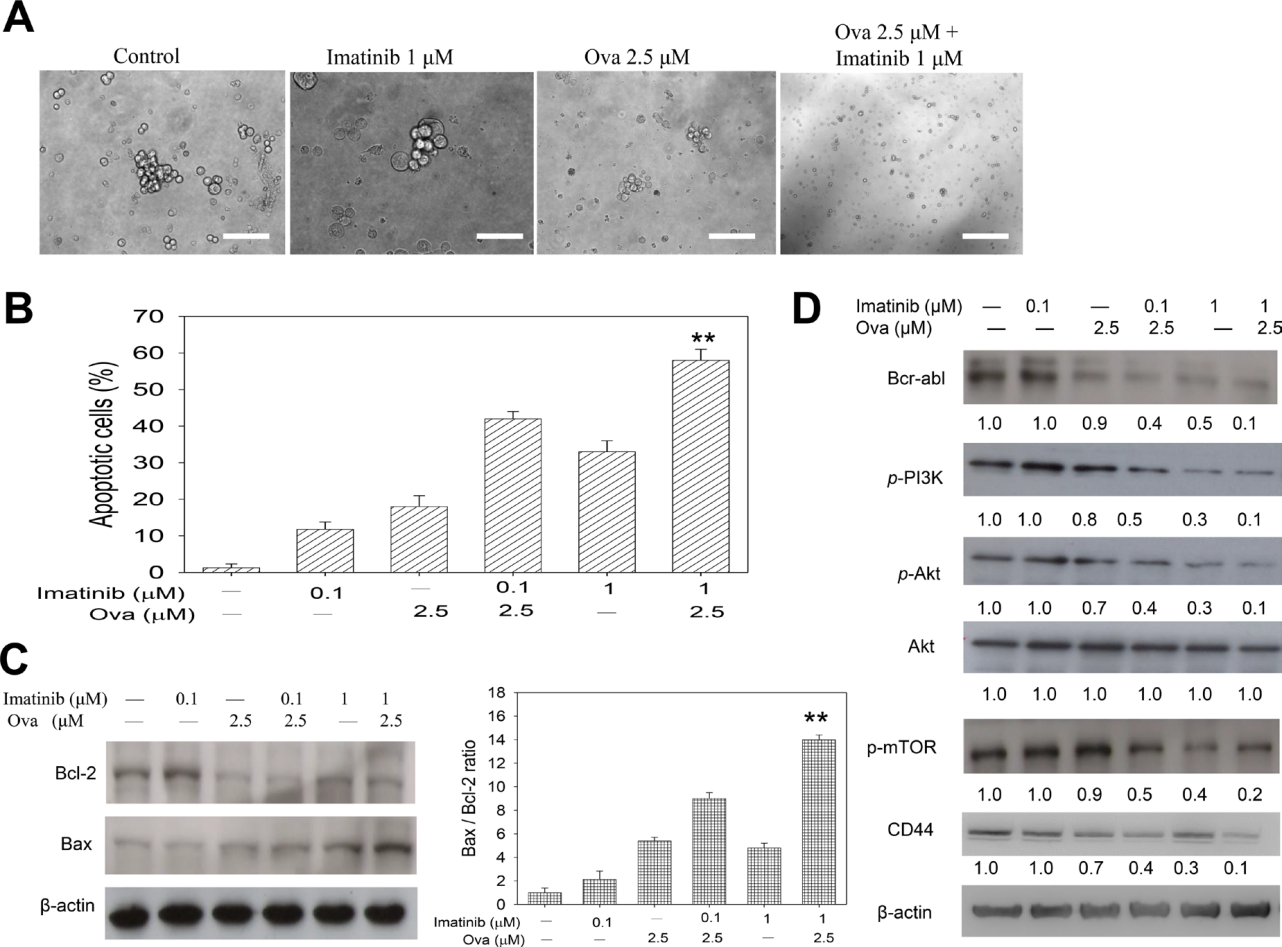
Diterpenoid ovatodiolide alone or in combination with Imatinib induce apoptotic cell death in CD34^+^CD38^−^ CML cells by dysregulating the PI3K/AKT/mTOR pathway (**A**) Tumorspehere formation assay for analysis of the effect of 1 μM Imatinib and/or 2.5 μM Ova treatment on the ability of CD34^+^38^−^ K562 cells to form tumorspheres. (**B**) Bar chart showing how treatment with 0.1–1 μM Imatinib and/or 2.5 μM Ova induce apoptosis in tumorspheres derived from CD34^+^38^−^ K562 cells. (**C**) Western blot analysis of Bcl-2 and Bax proteins expression level after treatment with 0.1–1 μM Imatinib and/or 2.5 μM Ova (left panel), and a graphical representation of the Bax/Bcl-2 ratio (right panel). (**D**) Western blot analysis of the effect of 0.1–1 μM Imatinib and/or 2.5 μM Ova on the expression levels of Bcr-abl, p-PI3K, p-Akt, Akt, p-mTOR, and CD44 proteins. β-actin was used as loading control. ^*^P < 0.05, ^**^P < 0.01, ^***^P < 0.001.

Having shown that Ova potentiates the anti-proliferative potential of Imatinib in the CD34^+^/CD38^−^ hCSC-like cells and disrupt their Bcr-Abl signaling, we investigated the underlying molecular mechanism. Firstly, since tumorspheres represent *in vitro* CSC models, to corroborate the previous data, we examined the effect of Ova and/or IM on the ability of the CD34^+^/CD38^−^ cells to form tumorspheres. Our data indicated that while 1 μM IM had no apparent effect on the tumorsphere formation ability of the CD34^+^/CD38^−^ hCSC-like cells compared to their unsorted counterparts, 2.5 μM Ova alone or in combination with 1 μM IM significantly inhibited the ability of the CD34^+^/CD38^−^ hCSC-like cells to form tumorspheres (Figure 3A). Then using the apoptosis assay, we demonstrated that while 0.1 μM IM induced apoptosis in 13%, when combined with 2.5 μM Ova, apoptotic cells increased to 44%, a 3.4-fold increase compared to IM alone. In same vein, apoptosis was induced in 33% or 58% of cells treated with IM alone or in combination with Ova, respectively, showing a 25% increase in the apoptotic cell population (Figure 3B and Supplementary Figure 1). This data was corroborated by the up-regulation of the pro-apoptosis marker, Bax, in the CD34^+^/CD38^−^ hCSC-like cells treated with 2.5 μM Ova alone or in combination with 0.1 or 1 μM IM, compared with IM alone or untreated control groups, as well as a down-regulation in the anti-apoptosis marker, Bcl-2 when treated with Ova alone or in combination with IM, compared with the IM alone or untreated control groups (Figure 3C). In addition, we demonstrated that this Ova-mediated increased induction of apoptosis and Bax/Bcl-2 ratio was correlated with dose-dependent significant reduction in the expression levels of Bcr-Abl, p-PI3K, p-Akt, p-mTOR and CD44 (Figure 3D and Supplementary Figure 2). It is notable that this inhibitory effect of Ova alone or in combination with IM in CD34^+^ hCSCs is similar to that elicited by silencing the gene that encodes for the p110a catalytic sub-unit of PI3K, PIK3CA, using a short interfering RNA (siPIK3CA) (Supplementary Figure 3). Similarly, Ova treatment was upregulates *hsa-miR-155* expression, while concurrently dysregulating the PI3K/mTOR signaling axis in the KU812 cells (Supplementary Figure 4). These data indicate that Ova by deregulating the PI3K/AKT/mTOR signaling pathway, increases the Bax/Bcl-2 ratio, induce cell death signals and lead to increased apoptosis of the CD34^+^/CD38^−^ hCSC-like cells.

### Ovatodiolide suppress CD34^+^/CD38^−^ hCSCs via epigenetic modulation of the PI3K/mTOR signaling axis

**Figure 4:**
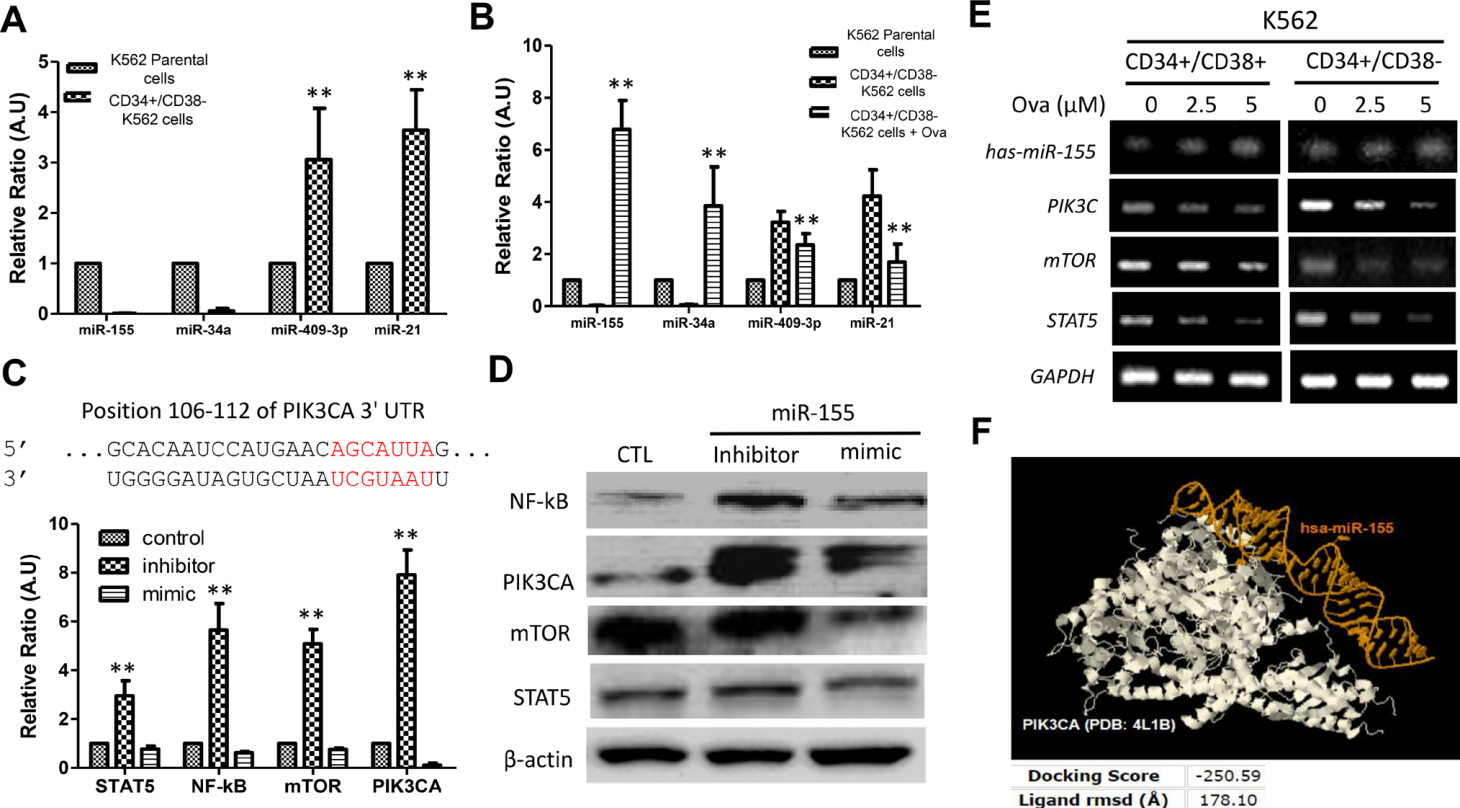
Diterpenoid ovatodiolide suppress CD34^+^/CD38^−^ hCSCs via epigenetic modulation of the PI3K/mTOR signaling axis (**A**) Comparative analysis of miR-155, miR-34a, miR-45-3p and miR-21 expression in CD34^+^38^−^ or parental K562 cells using RT-PCR. (**B**) The effect of Ova treatment on the expression of miR-155, miR-34a, miR-45-3p and miR-21 in CD34^+^38^–^ K562 cells, compared to the untreated CD34^+^CD38^–^ or parental K562 counterparts. (**C**) Mapping of the 5’ UTR of miR-155 to the 3’ UTR of the PIK3CA by TargetScan (upper panel). The differential effect of exposure to miR-155-5p inhibitor or mimic on the Stat5, NF-kB, mTOR and PI3KCA mRNA level in CD34^+^38^–^ K562 cells, compared with control K562 cells (lower panel). (**D**) Western blot analysis of the changes in NF-κB, PIK3CA, mTOR, and Stat5 protein expression level in CD34^+^38^−^ K562 cells in response to miR-155 inhibitor or mimic, in comparison to the control group. β-actin was used as loading control. (**E**) RT-PCR analysis of the effect of 2.5 µM–5 µM Ova treatment on the expressions of hsa-miR-155, PIK3CA, mTOR, and STAT5 in CD34+ K562 cells, compared to their untreated counterparts. GAPDH was used as loading control. (**F**) Molecular docking to demonstrate direct interaction between chain B8 of hsa-miR-155 and chain A of PI3KCA with a docking score of -250.59, and a ligand rmsd of 178.10 Å. PIK3CA, in white color. hsa-miR-155 in golden yellow color. RMSD, root-mean-square deviation, is the measure of the average distance between the atoms (usually the backbone atoms) of superimposed molecules.

**Figure 5:**
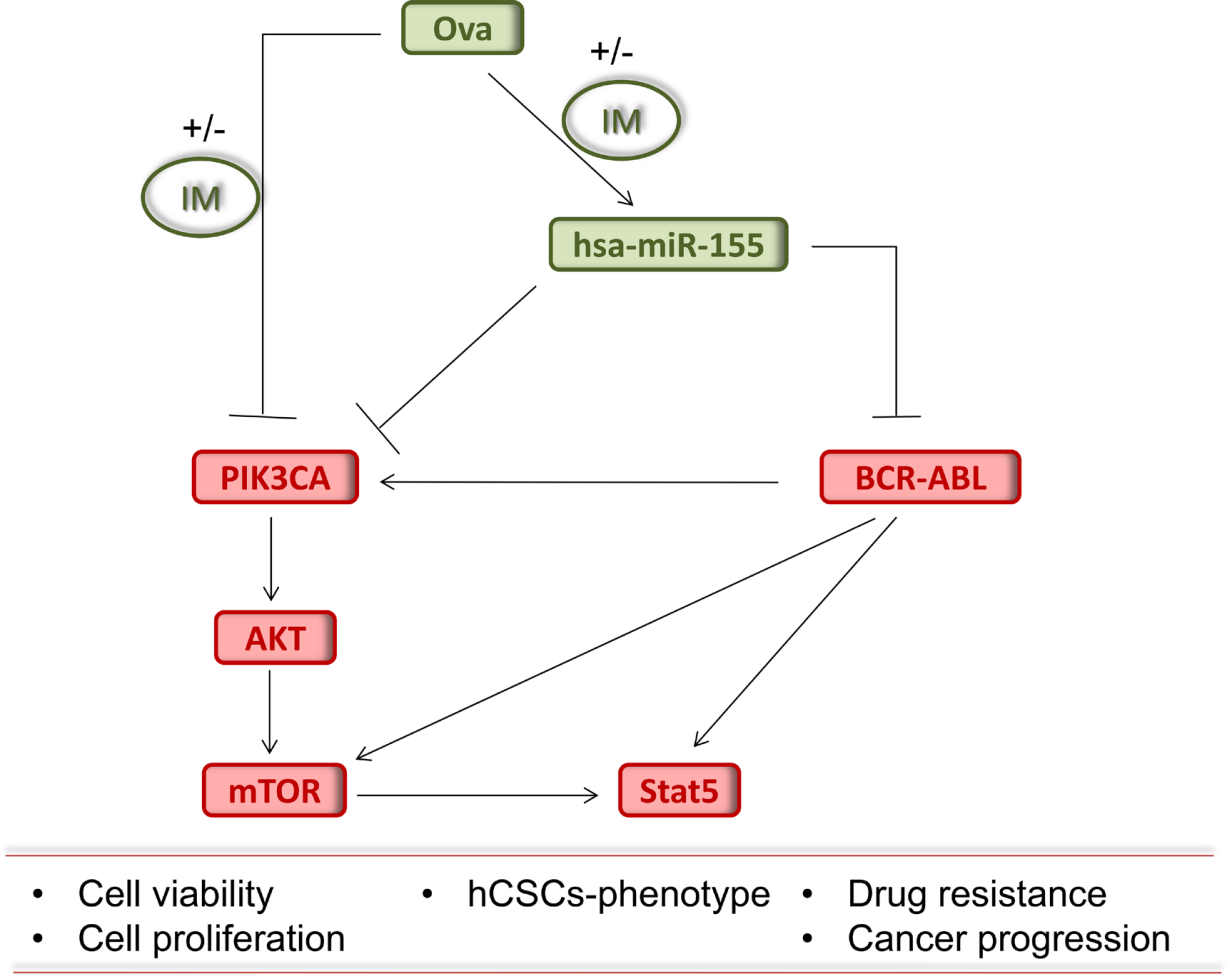
Pictorial Abstract Schematic summary showing that Ova with or without IM inhibits the growth of chronic myeloid leukemia stem cells by up-regulating hsa-miR-155, suppressing the BCR-ABL signaling and dysregulating the PI3K/AKT/mTOR pathway.

Based on the demonstrated critical role of miRNAs in erythropoiesis, granulopoiesis, monocytopoiesis, T-lymphocyte development, and the implication of altered miRNA expression in hematologic cancers [20], we analyzed the miRNA expression profile in CML hCSCs and unsorted K562 parental cells using RT-PCR. Our RT-PCR results showed that miR-155 and miR-34a were significantly down-regulated while miR-409-3p and miR-21 were conversely up-regulated in the CD34^+^/CD38^−^ hCSCs compared to the unsorted K562 parental cells (Figure 4A). Further, we demonstrated that treatment with 2.5 μM Ova could reverse this expression trend by significantly increasing the expression of miR-155 and miR-34a while inhibiting the expression of miR-409-3p and miR-21 in the Ova-treated CD34^+^/CD38^−^ hCSCs compared to their untreated CD34^+^/CD38^−^ or unsorted K562 parental counterparts (Figure 4B). By using TargetScan [22], we carried out genomic mapping of miR-155, and established that the 5′UTR of miR-155 binds to the position 106-112 of the 3′UTR of PIK3CA (Figure 5C, *upper panel*), which is the gene that encodes for the p110a catalytic sub-unit of PI3K to induce transcriptional repression of PI3K and by inference deregulate the PI3K/AKT/mTOR, as shown in the RT-PCR data where treatment with miR-155 mimic down-regulated the mRNA expression of Stat5, NF-kB, mTOR, and PIK3CA, while miR-155 inhibitor up-regulated their expression (Figure 4C, *lower panel*). This data was corroborated at the protein level by western blot analysis data (Figure 4D) and data from the RT-PCR analyses showing that treatment with 2.5μM and 5 μM Ova, increased the expression of hsa-miR-155 in CD34+ K562 cells, with corresponding upregulation in the expressions of PIK3CA, mTOR, and STAT5 (Figure 4E). The molecular docking to demonstrate direct interaction between chain B8 of hsa-miR-155 and chain A of PI3KCA with a docking score of -250.59, and a ligand RMSD of 178.10 Å (Figure 4F). These findings indicate that Ova effectively suppress CD34^+^/CD38^−^ hCSCs via a miR155-mediated epigenetic modulation of the PI3K/mTOR signaling axis.

## DISCUSSION

CML hCSCs constitute a small population of CML cells which play a critical role in the initiation and maintenance of CML [3, 4, 5]. In the present study, we identified that CD34^+^/CD38^−^ CML cells harboring the Bcr-Abl fusion gene, were enriched with Oct4, Sox2 and CD133 (Figure 1). The identification of the over-expression of these CSC-specific markers in the CD34^+^/CD38^−^ CML cells, has been associated with tumor aggressiveness, metastasis, resistance to treatment and tumor recurrence [1, 3], thus defining an enhanced hCSC-like phenotype. This is consistent with the seminal findings of initial studies by Bonnet and Dick’s team on leukemia wherein flow cytometry-based sorting was used to isolate a subpopulation of leukemic cells with the CD34^+^/CD38^−^ cell surface phenotype identified in bone marrow samples of acute myeloid leukemia (AML) patients and the inoculation of immune-compromised mice with 5,000 of these CD34^+^/CD38^−^ cells resulted in the development of leukemia in the mice models, thus, this small subset of leukemia cells with inherently greater stem cell self-renewal potential, compared to other leukemia or normal adult bone marrow cells, was then referred to as CSCs [23, 24].

Thus we worked on the premise that targeting these aggressive self-renewing Bcr-Abl^+^ CD34^+^/CD38^−^ hCSCs could be an effective strategy for the eradication of CML. Our study showed that though IM is a selective inhibitor of the t(9;22) chromosomal translocation-based BCR-ABL tyrosine kinase, unfortunately, its therapeutic effect was limited in scope and efficacy, especially against the CD34^+^/CD38^−^ hCSCs (Figure 2). Interestingly, this is not an isolated finding, as it is corroborated by another study which identified that despite the significant cytogenetic and hematological response observed in CML patients treated with IM, the presence of minimal residual disease detectable by sensitive quantitative -PCR, was inevitable, thus demonstrating that quiescent CML hCSCs were insensitive to STI571 or IM by using a FACS -based assay [25]. In the light of this observed innate reduced sensitivity of the CD34^+^/CD38^−^ hCSCs to the standard of care, IM, in the present study, we employed a new approach to eradicate these primitive quiescent Bcr-Abl^+^ CD38^−^ cells. We found that Ova, a bioactive macrocyclic diterpenoid isolate of Anisomeles indica (L.) Kuntze, with demonstrated anti-CSCs activity in solid tumor [12, 13] exhibited significantly inhibited the viability, Bcr-Abl signaling, and hCSC-like phenotype of these CD34^+^/CD38^−^ cells, and most importantly, we showed that Ova enhances the toxicity of IM across all CML cell populations, CD3^4^+/CD38^−^ inclusive (Figures 2 and 3). This anti-hCSCs activity of Ova in CML cells is consistent with previous results obtained in solid tumors, such as in the triple negative breast cancer (TNBC), where exposure to Ova increased the sensitivity of doxorubicin -resistant TNBC cells to doxorubicin, and induced the loss of CSC-like phenotype in the TNBC cells, as demonstrated by significant dissolution and necrosis of formed mammospheres [12], as well as in colon cancer, where treatment with Ova suppressed the expression of YAP1, inhibited M2 macrophage polarization, and prevented the generation of CSC-like colonospheres [26]. Our findings with Ova have clinical implications as these identified IM-insensitive hCSCs also exist *in vivo* as evidenced by the persistence of about 20% Bcr-Abl^+^ CD34^+^ and long-term culture-initiating cells in patients who achieved a complete cytogenetic response [27], low incidence of remission among IM-treated CML patients, and tumor recurrence when IM therapy is halted, often to blast crisis [28].

The therapeutic activity of Ova in the Bcr-Abl^+^ CD34^+^/CD38^−^ hCSCs was also shown to inhibit the phosphorylation of Stat5, up-regulate MDR and deregulate the PI3K/AKT/mTOR signaling pathway. During progression of CML, several signaling pathways become activated in cancer cells, however, many converge into principal downstream signaling networks, such as, the PI3K and STAT pathways which are very critical in the evolution of drug-resistant CML. Deregulating the activation of their components, such as Akt or Stat5 has been associated with curative efficacy in CML, suggesting that they are probable drivers of oncogenesis in the drug-resistant Bcr-Abl^+^ CD34^+^/CD38^−^ hCSCs [29]. This agrees with the demonstrated involvement of the Bcr-Abl tyrosine kinase with activation of several signaling pathways, such as the mitogen-activated protein kinase (MAPK), c-jun N-terminal kinase (JNK/SAPK), Ras small GTPase, nuclear factor kappa-light-chain-enhancer of activated B cells (NF-kB), signal transducers and activator of transcription (STAT), and phosphoinositide 3-kinases (PI3K), thus, promoting cell viability, proliferation and reduced apoptosis [30]. Currently, we are investigating Ova’s *in vivo* function using xenograft mouse model. Our preliminary data indicated that Ova treatment significantly suppressed tumorigenesis initiated by inoculating CD34+/CD38^−^ cells (data not shown). Further investigation was set to determine the potential synergistic effect of using Ova and Imatinib *in vivo*. This information will provide insights for designing future clinical trials for the treatment of drug-resistant CML patients.

Finally, we identified miRNAs that are deferentially expressed in CD34^+^/CD38^+^, CD34^+^/CD38^−^ and unsorted K562 cells, namely, miR-409-3p and miR-21 which are up-regulated and miR-34a and miR-155 which are down-regulated; in addition noting that Ova reverses this expression profile, such that after Ova treatment, expression levels of miR-409-3p and miR-21 are markedly reduced, while that of miR-34a and miR-155 are significantly enhanced, with associated down-regulation of Stat5, NF-kB, m-TOR and PIK3CA (Figure 4). These findings are in agreement with those published earlier demonstrating that miR-31, miR-155, and miR-564 are down-regulated in CML cells [31], creating a therapeutic window wherein the molecular or therapeutic (as in our case using Ova) enhancement of miR-155 expression and/or activity could play a critical role in reversing the oncogenic effect of the activated Stat5 and the PI3K/AKT/mTOR signaling pathway.

In conclusion, our studies identify a novel miR-155-dependent mechanism of regulating the aberrant oncogenic activity of the Stat5 and PI3K/AKT/mTOR signaling in the persistent IM-resistant the Bcr-Abl^+^CD34^+^/CD38^−^ hCSCs, which are major culprits in the altered growth, drug resistance, and recurrence observed in CML patients (Figure 5). Our study adds another layer to cumulative evidence of miRNA involvement and the possibility of targeting miRNA-modulated signaling pathways to improve the clinical outcome of CML patients.

## MATERIALS AND METHODS

### Drugs and chemicals

Imatinib mesylate (IM, SML1027 SIGMA; ≥98% HPLC) purchased from Sigma Aldrich Co. (St. Louis, MO, USA). Stock solution of 1 μM dissolved in PBS was stored at −4°C. Diterpenoid ovatodiolide was prepared as the previous report [32]. Ova was dissolved in DMSO and further diluted in sterile culture medium immediately prior to use. Gibco^®^ RPMI 1640, Trypsin/EDTA, dimethyl sulfoxide (DMSO), phosphate buffered saline (PBS), sulforhodamine B (SRB) medium, Acetic acid and TRIS base were also obtained from Sigma Aldrich Co.

### Cell lines and culture

The human CML cell line K562 was obtained from the American Type Culture Collection (ATCC. Manassas, VA., USA). Cells were cultured in Dulbeco’s modified Eagle’s medium (DMEM, Invitrogen Life Technologies, Carlsbad, CA), supplemented with 10% fetal bovine serum (FBS) and 1% penicillin/streptomycin (Invitrogen, Life Technologies, Carlsbad, CA) and incubated at 37°C in 5% humidified CO_2_ incubator. The K562 cells, their CD34^+^/CD38^−^ and CD34^+^/CD38^+^ variants were treated with different concentrations of Ova and/or IM.

### Fluorescence-activated cell-sorting analysis using flow cytometry

We isolated CD34^−^, CD34^+^, CD34^+^CD38^−^, and CD34^+^CD38^+^ CML K562 cells by performing the fluorescence-activated cell-sorting (FACS) assay as previously described [33], and defined the quadrants by fluorescein isothiocyanate (FITC) and PE-labeled isotype controls. Further, we obtained defined numbers of CML cells for reverse transcription polymerase chain reaction (RT-PCR) analyses following a previously described protocol [34].

### Western blot analysis

By using the 10% SDS-PAGE gel, 15 μg total protein samples was fractionated by electrophoresis and transferred to a Polyvinylidene fluoride (PVDF) membrane in the Bio-Rad Mini-Protein electro-transfer system (Bio-Rad Laboratories, Inc, CA, USA). Incubation of the membranes in 5% skimmed milk - Tris-buffered saline with Tween 20 (TBST) for 1 h to block non-specific binding, was followed by incubation overnight at 4°C with the antibodies against Bcr-Abl (1 : 1000, Cell Signaling Technology), Oct4 (1 : 1000, Santa Cruz), Sox2 (1 : 1000, Cell Signaling Technology), p-CrkL (1 : 1000, Cell Signaling Technology), Stat5 (1 : 1000, Santa Cruz), p-Stat5 (1 : 1000, Santa Cruz), MDR (1 : 1000, Cell Signaling Technology), CD133 (1 : 1000, Cell Signaling Technology), pAkt (1 : 1000, Santa Cruz), Akt (1 : 1000, Santa Cruz), p-PI3K (1 : 1000, Cell Signaling Technology), PI3K (1 : 1000, Cell Signaling Technology), p-mTOR (1 : 1000, Cell Signaling Technology), CD44 (1 : 1000, Santa Cruz), and β-actin (1 : 500, Santa Cruz). The membranes were then washed and incubated in appropriate horseradish peroxidise (HRP)-conjugated secondary antibodies for 1 h at room temperature, then washed with PBS 3 times. Protein bands were then detected in the enhanced chemiluminescence (ECL) detection system (Thermo Fisher Scientific Inc, Waltham, MA, USA) and quantification was done using the ImageJ software.

### Culturing of cells into tumorspheres

After sorting the cells, the unsorted, CD34^+^/CD38^–^, and CD34^+^/CD38^+^ cells were seeded at 1000 cells per well in 6-well non-adherent plates (Corning Inc., Corning, NY) in DMEM/F12 medium supplemented with bFGF (20 ng/mL, Invitrogen, Carlsbad, CA), B27 supplement (Invitrogen, Carlsbad, CA), and EGF (20 ng/mL, Millipore, Bedford, MA). Cells were cultured for 10–12 days, and the formed tumorspheres were counted using inverted phase contrast microscopy.

### Reverse-transcription polymerase chain reaction (RT-PCR)

Total RNA was extracted from the CML cells using Trizol reagent according to the manufacturer’s instructions, and reverse-transcribed using the first-strand cDNA Synthesis Kit (Fermentas UAB, Vilnius, Lithuania) following the manufacturers’ instructions. RT-PCR was carried out with 50 μL of reaction-mix containing 1 μg cDNA template, 1 μM specific oligonucleotide primers, and 25 μL Taq mixtures containing 0.5 unit of Taq DNA polymerase. We separated PCR products by electrophoresis in 2% agarose gel.

### Sulforhodamine B cell viability assay

CD34^+^/CD38^−^, and CD34^+^/CD38^+^ cells seeded in triplicate per plate in 96-well plate were cultivated for 24 h, then incubated with Ova and/or IM for 48 h. The treated CD34^+^/CD38^−^, and CD34^+^/CD38^+^ cells were fixed with 10% Trichloroacetic acid (TCA), washed with ddH_2_O, and stained with 0.4% SRB (w/v) in 1% acetic acid. The unbound dye was removed by carefully washing with 1% acetic acid 4 times and the plates were air-dried. Bound SRB dye was then dissolved in 10 μM Trizma, and the absorbance was read at 570 nm wavelength in a microplate reader.

### Statistical analysis

Each experiment was performed at least 3 times in duplicates. All data represent means ± SD. Comparison between two groups was estimated using the 2-sided Student’s *t*-test, while the one-way analysis of variance (ANOVA) was used for comparison between 3 or more groups. *P*-value < 0.05 was considered statistically significant.

## SUPPLEMENTARY MATERIALS FIGURES


