# Supplementary material for: Ovatodiolide targets chronic myeloid leukemia stem cells by epigenetically upregulating hsa-miR-155, suppressing the BCR-ABL fusion gene and dysregulating the PI3K/AKT/mTOR pathway
Source: Oncotarget. 2017 Dec 14;9(3):3267–77. doi: 10.18632/oncotarget.23231 (PMC5790462; doi:10.18632/oncotarget.23231)
Supplement: Supplementary file 1 [file oncotarget-09-3267-s001.pdf]

Ovatodiolide targets chronic myeloid leukemia stem cells by epigenetically upregulating hsa-miR-155, suppressing the BCR-ABL fusion gene and dysregulating the PI3K/AKT/mTOR pathway

SUPPLEMENTARY MATERIALS

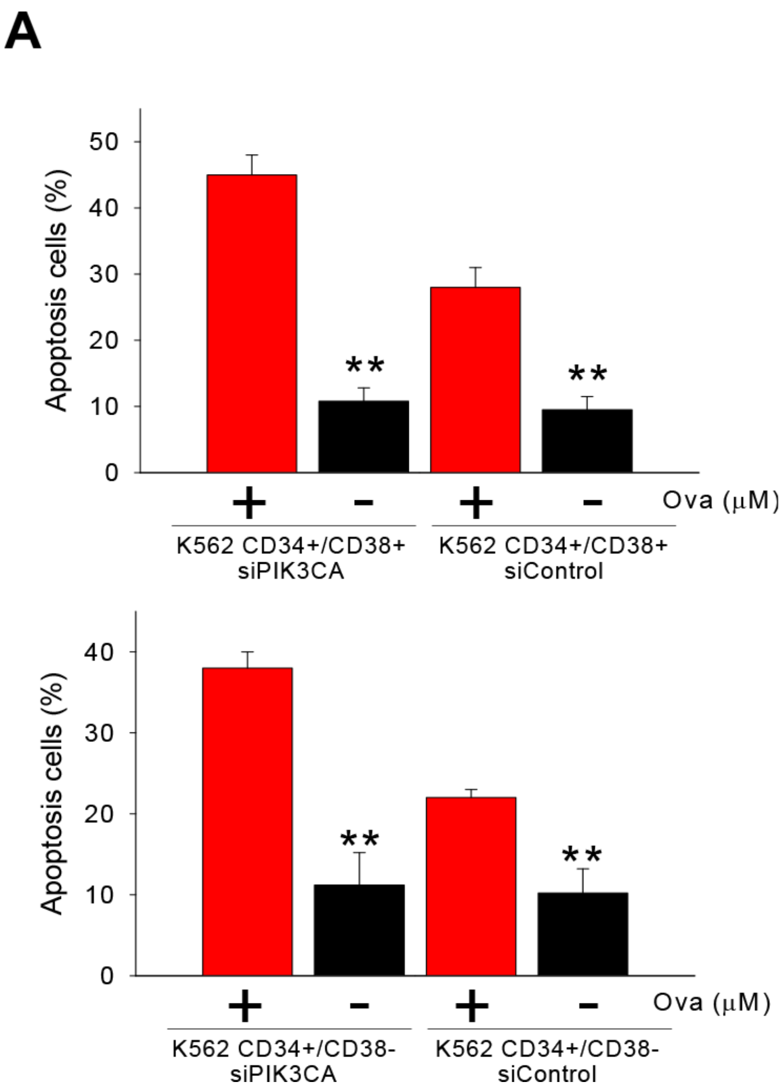

Supplementary Figure 1: Down-regulating PIK3CA expression has an antiproliferative potential and enhance apoptosis of $CD34^+$ CML cells. (**A**) Graphical representation of the apoptosis-inducing effect of 18 h treatment with 2.5 mM Ova in the absence or presence of siPIK3CA in $CD34^+/CD38^+$ (left panel) and $CD34^+/CD38^-$ (right panel) K562 cells. Cells tranfected with mock siRNA (siControl) served as control.

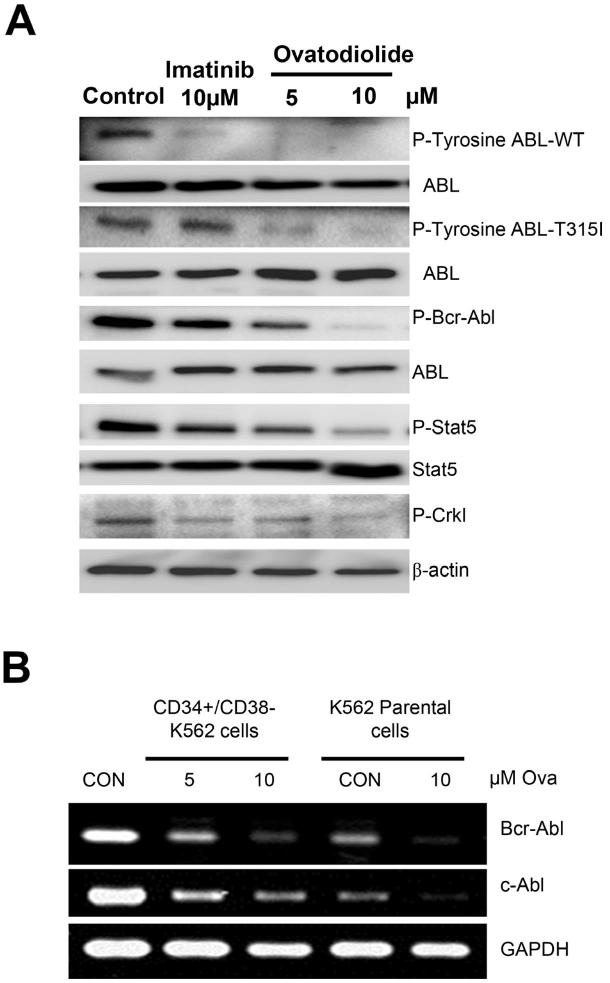

Supplementary Figure 2: Diterpenoid ovatodiolide alone or in combination with Imatinib disrupt Bcr-Abl signaling in $CD34^{+}CD38^{-}$ CML cells. (**A**) Western blot analysis of the effect of 10 µM Imatinib or 5–10 µM Ova on the expression levels of p-Abl, Abl, p-Abl-T3151, pBcr-Abl, p-Stat5, Stat5, and p-CrkL proteins in CD34+/$CD38^{-}$ cells, compared to untreated control cells. β-actin was used as loading control. (**B**) Changes in the expression levels of Bcr-Abl and c-Abl mRNA in $CD34^{+}$/$CD38^{-}$ and unsorted parental K562 cells, after treatment with 5–10 µM Ova. GAPDH was used as internal control.

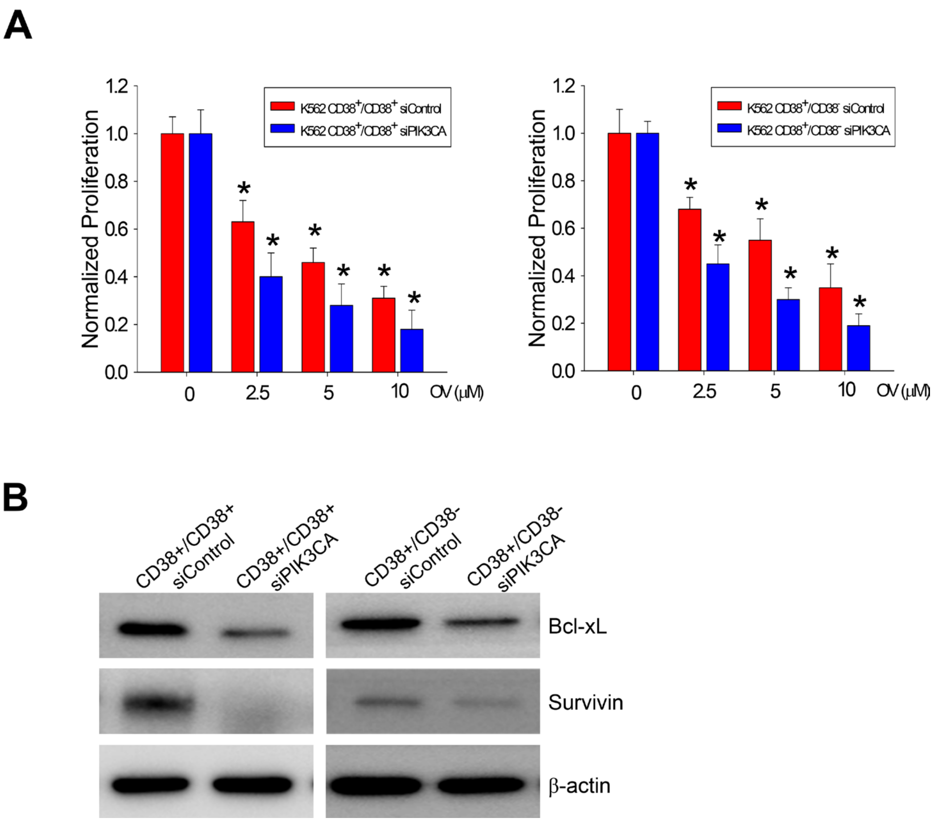

Supplementary Figure 3: Down-regulating PIK3CA expression has an antiproliferative potential and enhance apoptosis of CD34$^+$ CML cells. (**A**) The effect of 24 h Ova treatment on the proliferation of CD34$^+$/CD38$^+$ (left panel) and CD34$^+$/CD38$^-$ (right panel) K562 cells evaluated by SRB assay. (**B**) Western blot analysis of the expression profile of Bcl-xL and Survivin proteins in CD34$^+$/CD38$^+$ and CD34$^+$/CD38$^-$ K562 cells transfected with PIK3CA siRNA, compared with mock-transfected control cells. β-actin was used as the loading control. $^*P < 0.05$, $^{**}P < 0.01$, $^{***}P < 0.001$.

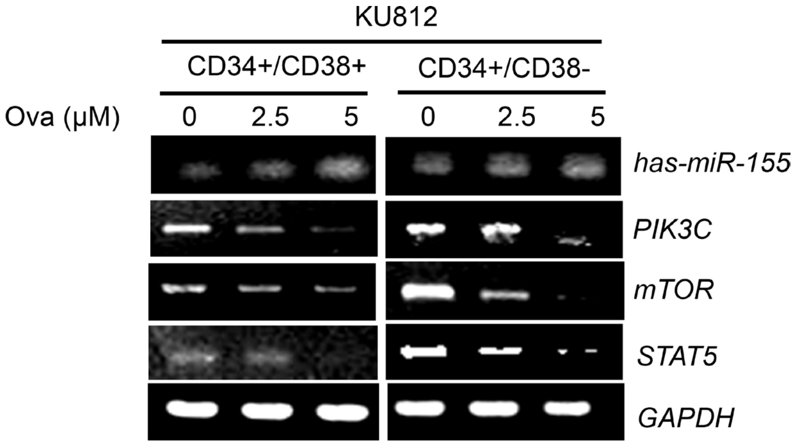

Supplementary Figure 4: Ova treatment upregulates *hsa-miR-155* expression, while concurrently dysregulating the PI3K/mTOR signaling pathway. RT-PCR analysis of the effect of 2.5 μM–5 μM Ova treatment on the expressions of *hsa-miR-155, PIK3CA, mTOR,* and *STAT5* in CD34+ KU812 cells, compared to their untreated counterparts. GAPDH was used as loading control.
